# An update on adaptive deep brain stimulation in Parkinson's disease

**DOI:** 10.1002/mds.115

**Published:** 2018-10-24

**Authors:** Jeroen G.V. Habets, Margot Heijmans, Mark L. Kuijf, Marcus L.F. Janssen, Yasin Temel, Pieter L. Kubben

**Affiliations:** ^1^ Departments of Neurosurgery Maastricht University Medical Center Maastricht The Netherlands; ^2^ Department of Neurology Maastricht University Medical Center Maastricht The Netherlands; ^3^ Department of Clinical Neurophysiology Maastricht University Medical Center Maastricht The Netherlands; ^4^ School of Mental Health and Neuroscience Maastricht University Medical Center Maastricht The Netherlands

**Keywords:** adaptive, closed‐loop, deep brain stimulation, Parkinson's disease, stimulation paradigms

## Abstract

Advancing conventional open‐loop DBS as a therapy for PD is crucial for overcoming important issues such as the delicate balance between beneficial and adverse effects and limited battery longevity that are currently associated with treatment. Closed‐loop or adaptive DBS aims to overcome these limitations by real‐time adjustment of stimulation parameters based on continuous feedback input signals that are representative of the patient's clinical state. The focus of this update is to discuss the most recent developments regarding potential input signals and possible stimulation parameter modulation for adaptive DBS in PD. Potential input signals for adaptive DBS include basal ganglia local field potentials, cortical recordings (electrocorticography), wearable sensors, and eHealth and mHealth devices. Furthermore, adaptive DBS can be applied with different approaches of stimulation parameter modulation, the feasibility of which can be adapted depending on specific PD phenotypes. Implementation of technological developments like machine learning show potential in the design of such approaches; however, energy consumption deserves further attention. Furthermore, we discuss future considerations regarding the clinical implementation of adaptive DBS in PD. © 2018 The Authors. *Movement Disorders* published by Wiley Periodicals, Inc. on behalf of International Parkinson and Movement Disorder Society.

Conventional deep brain stimulation (cDBS) of the subthalamic nucleus (STN) or the globus pallidus internus (GPi) is an established treatment for advanced stage Parkinson's disease (PD). Although cDBS improves the motor symptoms of PD in both the short and long term, it is not without limitations.^1^, ^2^ Stimulation‐induced side effects such as dysarthria,^3^ imbalance, and dyskinesia can occur and often require regular adjustments in stimulation, especially in the first phase after surgery.^4^ Moreover, cDBS has limited battery life. These limitations have led to development and expanding scientific interest in closed‐loop, responsive, or adaptive DBS (aDBS; Fig. 1). For consistency reasons, only the term aDBS will be used.

**Figure 1:**
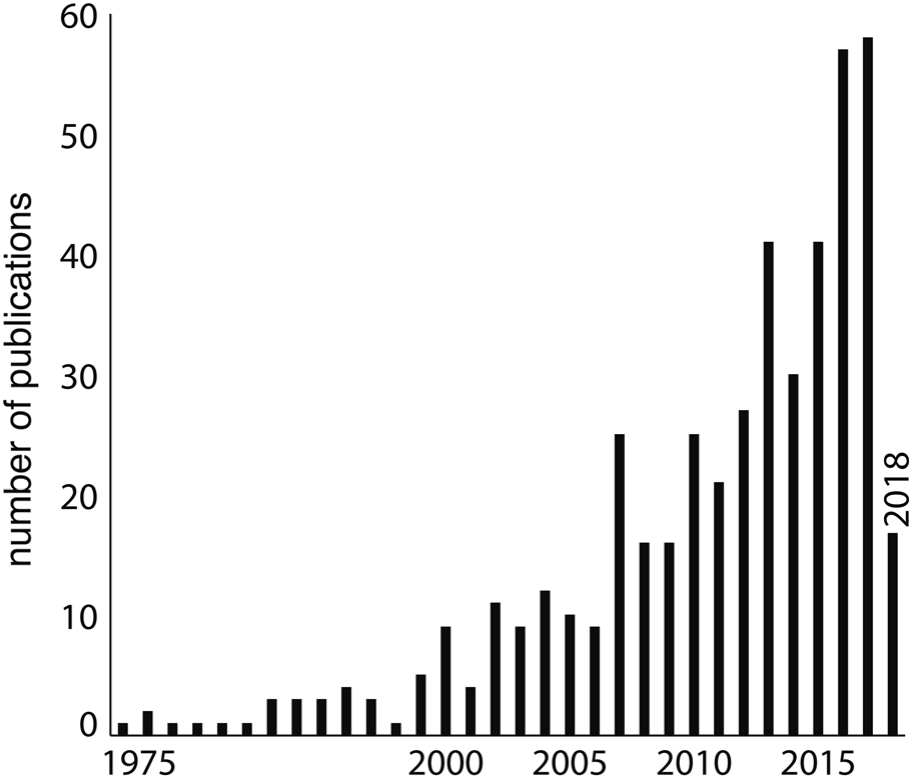
Yearly number of publications on aDBS in PD, searched on PubMed on 5‐3‐2018, using search command: [(parkinson*) AND (adaptive OR (closed loop) OR (closed‐loop) OR responsive) AND (dbs OR stimulation)].

In cDBS, stimulation parameters are traditionally programmed and evaluated by a clinician during outpatient visits. If necessary, stimulation parameters are adjusted, and patients can perform minor changes within preset ranges themselves later. The goal of aDBS is to optimize this process further and automatically adapt stimulation parameters to the fluctuating clinical state of the patient, where, in theory, stimulation is given only when necessary. As such, aDBS may generate fewer side effects attributed to the possible decrease in energy given. In addition, although more power may be needed for data processing, the required battery consumption for stimulation potentially decreases and could result in increased battery longevity. Clinical proof‐of‐concept studies have already shown beneficial results using electrophysiological and/or wearable sensor recordings as feedback signals for aDBS in PD.^5^, ^6^ The next step is to confirm whether such an approach continues its efficacy in the long term and discuss new issues on the design of aDBS.

Development of a valid aDBS system in PD faces major challenges such as creating suitable input and processing input signals into beneficial output. In the following sections, we present an update, future needs and possibilities for input signals, and stimulation paradigms for aDBS in PD. Much of the technological and clinical knowledge and experience discussed here also relates to the use of aDBS in other fluctuating neurological and psychiatric diseases, such as essential tremor (ET), dystonia, epilepsy, Tourette's syndrome, and obsessive‐compulsive disorder.

## Potential Input Signals for aDBS

**Figure 2:**
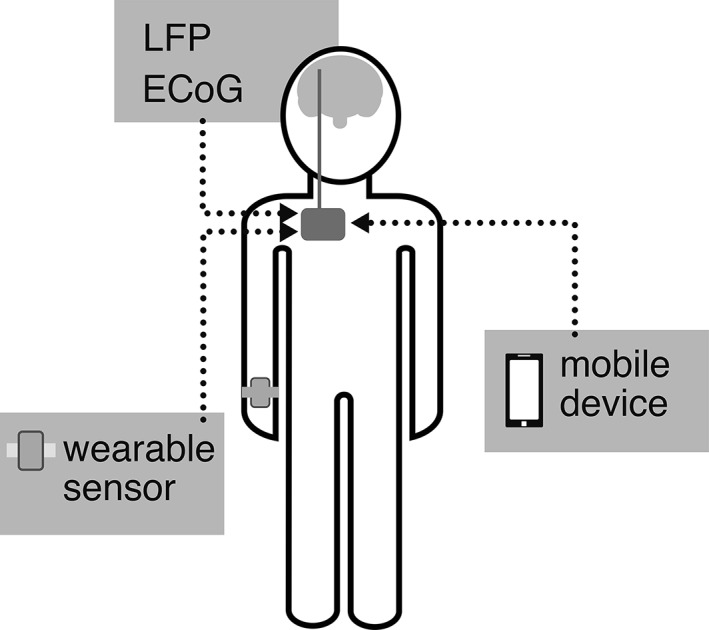
Schematic overview of the most used possible input signal origins for aDBS in PD. Sensors can also be worn on different locations, for example, the chest, legs, or fingers.

**Figure 3:**
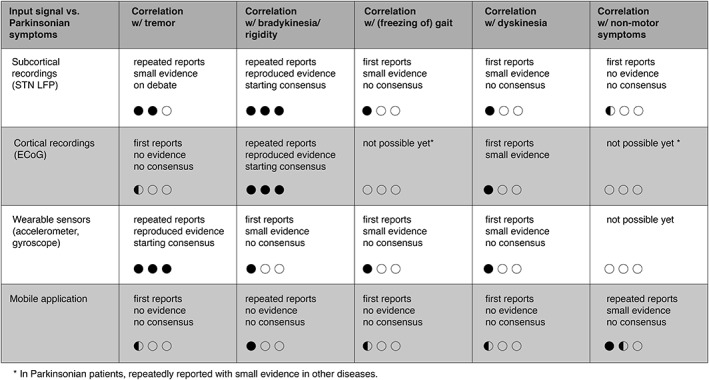
Overview of published evidence of the feasibility of different input signals regarding different parkinsonian symptoms for aDBS in PD. All input signals are scored on three categories per symptom. For each category 0, 0.5, or 1 bullet is given and the sum of them is visualized. The first line indicates the amount of publications: not possible yet (0), first reports (0.5), and repeated reports (1). The second line indicates the quality of reported evidence: no evidence (0), small evidence (0.5), and reproduced evidence (1). The third line indicates the amount of consensus on the use of an input signal for a symptom: no consensus (0), on debate (0.5), and starting consensus (1).

To develop a valid aDBS system, robust input signals representing the main PD symptoms are needed (Fig. 2). Symptoms vary from patient to patient, and therefore the suitability of input signals differs for individual patients (Fig. 3). Furthermore, the necessity of supplementary (non‐)invasive implants or devices, as well as additional processing and computational demands, should be taken into consideration when comparing input signals. A comprehensive and concise overview of rationales and basic principles regarding potential input signals has recently been reviewed elsewhere.^7^ We will elaborate further on previous work by discussing up‐to‐date progress and the remaining challenges regarding potential input signals for aDBS in PD.

## aDBS Based on Electrophysiological Recordings

### Basal Ganglia Recordings

New‐generation DBS pulse generators can record local field potentials (LFPs), which have been correlated with clinical symptoms in several studies. For example, decreased beta band (8‐35 Hz) activity in the STN by dopaminergic medication and/or DBS has been correlated with improved akinesia, bradykinesia, and rigidity,^8^, ^9^ but not with tremor.^10^, ^11^, ^12^ However, others found a correlation between STN‐LFP recordings and tremor.^13^, ^14^ Furthermore, freezing‐of‐gait periods^15^ and differentiation between speech and movement activities^16^ can be detected using STN‐LFPs. Such differentiation of clinical indications underlines the potential of STN‐LFP recordings as promising input signals, with the added benefit of not requiring additional implants or equipment compared to cDBS.^17^

A number of proof‐of‐concept studies using beta‐LFPs to modify aDBS have shown motor improvement,^6^, ^18^ less speech impairment,^19^ and less levodopa‐induced dyskinesia compared to cDBS,^20^ which suggests that this is a more efficient and effective method of stimulation. Moreover, a recent study demonstrated the feasibility and beneficial effects on motor symptoms of aDBS over the course of 8 hours in akinetic‐rigid PD patients.^21^ Previous studies had already shown that aDBS was applicable and effective in a freely moving^22^ and a chronically implanted PD patient.^23^

Nevertheless, beta‐LFPs in the STN are not (easily) detectable in all patients,^8^ although this long‐standing assumption has been contradicted recently.^24^ Second, changes in beta‐LFPs do not clearly capture *all* main symptoms of PD. For example, the relationship with tremor is debated. Although other STN‐LFP signals like theta‐band (3‐8 Hz) activity show promise in relation to tremor,^14^ additional input signals to monitor tremor might be needed.^24^, ^25^ Third, the clinical relevance or symptomatic contribution of high‐ versus low‐beta bands is a topic of discussion.^26^, ^27^ Last, alpha/beta‐band activity is influenced by daily life events such as rest tremor,^24^ voluntary movements,^28^, ^29^ movement artifacts during gait,^30^ different vigilance states and sleep,^31^ and aDBS itself,^32^ which makes the isolation of disease‐related signals difficult.

Despite these challenges, basal ganglia LFPs have been shown to function as a suitable input signal for aDBS. The main challenge to enable clinical use of LFPs is the development of standardized techniques that allow for automatic and validated interpretation of input signals. Therefore, further development of the hardware and software of aDBS systems is needed to acquire various frequency bands or additional input signals. Eventually, these sophisticated aDBS systems should better suit the difference in clinical needs between akinetic‐rigid and tremor‐dominant PD patients.

### Cortical Recordings

A hallmark of PD is pathological hyperactivity of the corticobasal pathways, which is attributed to dopamine denervation of the striatum and substantia nigra.^33^ This hyperactivity results in clinically identifiable cortical oscillations, which can be measured invasively by electrocorticography (ECoG) using a subdural grid. aDBS can utilize these cortical oscillations as an input signal. For instance, one study showed that GPi‐aDBS in nonhuman primates based on motor cortex (M1) beta‐activity resulted in beneficial effects on akinesia.^34^ Spatial‐specific attenuation of cortical beta‐hypersynchrony was also demonstrated in humans subsequent to STN‐DBS.^35^ Recent studies use phase‐amplitude coupling (PAC), whereby the amplitude of specific bandwidth oscillations is coupled to specific oscillation phases. In akinetic‐rigid PD patients, excessive M1‐beta‐gamma‐PAC decreased during STN‐DBS, parallel to a decrease of clinically assessed bradykinesia.^36^, ^37^ In contrast, in tremor‐dominant PD patients, excessive M1‐beta‐PAC decreased during rest tremor.^38^ Moreover, ECoG recordings showed potential to monitor dyskinesia,^39^ gait characteristics, such as walking duration and speed,^40^ and to perform speech recognition.^41^ Interpretation of cortical PAC values therefore requires differentiation between phenotypic manifestations.

This work led to use of a fully implanted ECoG‐based aDBS device in PD patients who experienced moderate dyskinesia despite optimized STN‐DBS therapy.^42^ The researchers adjusted stimulation voltage based on gamma‐band (60‐90 Hz) activity, which is related to dyskinesia. The clinical effect on bradykinesia and dyskinesia was maintained, while energy savings were ∼40%.

A remaining concern is the limited correlation with PD symptoms and PAC attenuation attributed to movement preparation and execution.^37^ Equal to beta‐LFP in the STN, cortical beta‐PAC is altered by DBS, which has implications for the analytical process.^43^ Moreover, the time‐frequency method used most often in PAC analysis might cause artifacts attributed to ignorance of the existence of both harmonic and nonsinusoidal neural dynamics in PD.^44^ Another concern is that implantation of subdural grids may be associated with increased risk of complications, such as hemorrhage and infection. As recently demonstrated, use of cortical PAC is promising because of its potential ability to decode movement and behavior. Therefore, further steps are warranted to integrate the analyzed information from PAC and develop analytic algorithms for different PD symptoms to perform aDBS based on cortical recordings in the whole PD spectrum.

### Surface Electromyography

For several decades, surface electromyography (sEMG) signals have been used in tremor detection and more recently in tremor prediction.^45^, ^46^, ^47^, ^48^ Therefore, sEMG is considered to be a potential input signal for aDBS for ET and tremor‐dominant PD. sEMG‐based aDBS was feasible, effective, and efficient in ET patients.^49^, ^50^, ^51^ Given that evidence of sEMG‐based bradykinesia and rigidity detection methods is limited,^52^, ^53^ sEMG should be combined with other input signals for akinetic‐rigid PD patients. Another major concern of sEMG‐based aDBS is potential loss of data quality attributed to the required self‐management of sEMG sensors by patients. Furthermore, signals must be processed and transmitted wirelessly to the pulse generator, which, in turn, may limit its battery life. To overcome these disadvantages, wireless sEMG sensors should be developed to withstand high contact impedances by using, for example, interchangeable patches to attach them to the skin or subcutaneous implantable EMG electrodes. However, the limited potential of sEMG as an input signal and the current progress in wearable sensor development seem to make sEMG impractical for aDBS in PD.

## aDBS Based on Neurochemical Recordings

As stated in previous work, development of aDBS based on neurochemical recordings is in an early phase.^7^ Artifact‐free neurochemical recordings were possible during DBS in rodents,^54^ and dopamine fluctuations depending on DBS were found.^55^ Therefore, neurochemical recordings were regarded to be a potential input signal for aDBS; however, the relationship between neurochemical recordings, PD symptoms, and DBS in humans has not been explored. Because no progress has been reported recently, limitations for clinical use of neurochemical feedback in aDBS remain substantial.

## aDBS Based on Wearable Sensors

Monitoring PD symptoms through wearable sensors, or “wearables,” containing accelerometers and/or gyroscopes has gained considerable interest, and important progress has been made in the last decade.^56^ Wearables are successful in predicting and detecting tremor^46^, ^48^, ^57^ and show promise in assessing freezing of gait,^58^ bradykinesia, and dyskinesia.^59^, ^60^, ^61^

Numerous studies based on tremor detection have supported the feasibility, effectiveness, and efficiency of wearables‐based aDBS.^5^, ^51^, ^62^ However, no other PD symptoms are yet detectable or implemented with wearable aDBS systems, and therefore the applicability for akinetic‐rigid PD patients is unclear.^62^

Application of wearables for aDBS will rely heavily on machine‐learning approaches for distinguishing symptoms from voluntary movements.^63^ Another concern is that patients will need to wear the sensors almost chronically. However, given that sensors are getting smaller and more aesthetically attractive, this might not be a problem for all. In addition, continuous assessment of PD symptoms at home using wearables does not affect health‐related quality of life.^64^ Last, signal processing and wireless data transmission may limit battery life of wearables and pulse generators.

To implement aDBS controlled by wearables, algorithms to monitor other cardinal motor symptoms than tremor need further development and clinical validation to expand the potential for akinetic‐rigid PD patients. For tremor‐dominant patients, clinical trials with longer follow‐up periods should be done to prove superiority compared to cDBS.

## aDBS Based on PD Monitoring Systems Including eHealth and mHealth Applications

Wearables and electrophysiological recordings disregard the subjective experience of motor symptoms and assessment of nonmotor symptoms. We believe that subjective experience of motor symptoms could improve the interpretation of objective motor symptom monitoring. Nonmotor symptoms are important for quality‐of‐life scores and might predict overall DBS outcomes.^65^ Electronic health (eHealth) and mobile health (mHealth) applications and telemonitoring concepts have been recently integrated into PD care and contain the aforementioned missing features.^66^, ^67^, ^68^, ^69^ Most of these developments are achieved in order to improve PD care and ensure its accessibility and cost‐effectiveness.^70^, ^71^ However, these developments also hold promise for aDBS.

A recent trial demonstrated that cDBS‐setting adjustment by telemonitoring was feasible.^72^ Introducing automated monitoring and increasing the frequency of DBS‐setting adjustment brings this concept close to (semicontinuous) aDBS. The lack of valid continuous PD monitoring tools led to development of multimodal PD monitoring systems. These systems include, for example, wearables and mobile applications and distinguish themselves from systems discussed above by adding assessments of cognition, speech, subjective disease burden, and active motor tasks. This potential was recently underlined by development of a smartphone application to capture symptom fluctuation during the day.^73^

Several recently initiated trials test the feasibility and clinical value of multimodal PD monitoring systems in the patient's home environment. So far, these systems aim to differentiate ON/OFF states by wearables and a diary,^74^ detect the need for changes in or improve adherence of pharmacological therapy,^75^, ^76^, ^77^ and monitor clinical well‐being in a holistic fashion.^78^, ^79^ Other systems aim to detect relevant neurophysiological biomarkers for home monitoring in order to improve postoperative DBS care^67^ and assess the effect of DBS parameter adjustments with wearables.^69^ Furthermore, feasibility of the experience sampling method is demonstrated among PD patients.^80^ This method collects subjective experiences of both motor and nonmotor symptoms multiple times a day during the flow of daily life.

The abovementioned studies show the feasibility of using multimodal monitoring systems among PD patients. We believe there might be a role for such multimodal PD monitoring systems in aDBS, because they have the potential to combine subjective assessments of burden and nonmotor symptoms with objective input signals. Especially during the initial postoperative phase, combining these input signals may be of great value for adjusting DBS. Feasibility of such a holistic approach should be explored further.

## Stimulation Parameter Modulation in aDBS

The process of collecting continuous data representing (non‐)motor symptoms is the first major challenge for developing an aDBS system for PD. A second major challenge is the design of a system that automates the complex reasoning and decision making currently achieved by clinicians, which will require more advanced and distinctive signal processing than is currently available. This challenge contains several issues, such as frequency of stimulation parameter adjustments, the nature of stimulation parameter adjustments, data transfer, data computation, and battery consumption.

Most aDBS research has so far focused on potential input signals, and therefore several issues regarding the design of stimulation parameter modulation are less well studied. In the following sections, we discuss the current progress and remaining challenges regarding stimulation parameter modulation in aDBS.

### Amplitude Modulation Approaches

**Figure 4:**
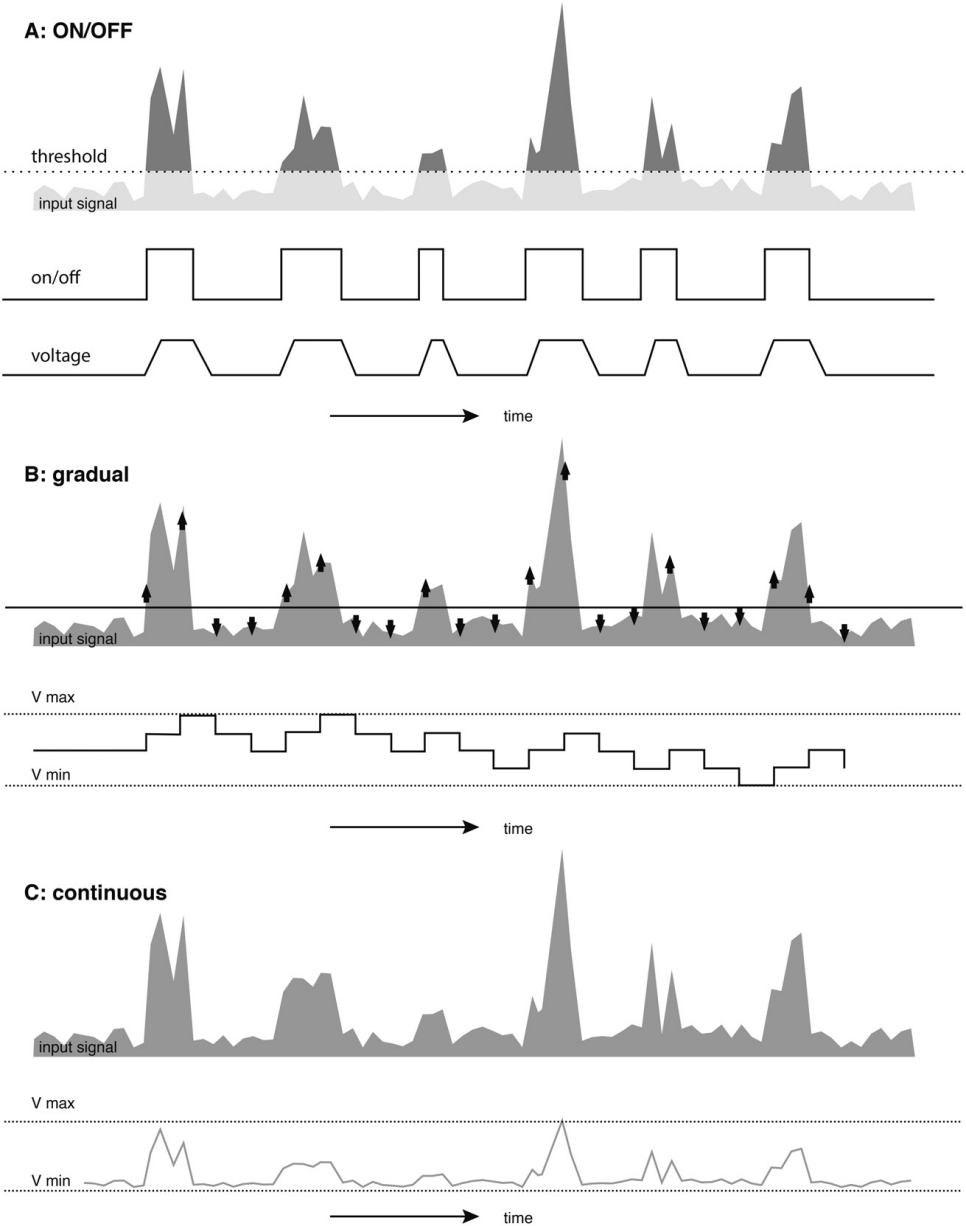
Schematic overview of different amplitude modulation paradigms used in aDBS in PD. (A) ON/OFF paradigm, which stimulates with ramping onset when input signals exceed a certain threshold. (B) Gradual paradigm, which increases or decreases stimulation amplitude stepwise when input signal exceeds or does not exceed a certain threshold respectively. (C) Continuous paradigm, which modifies stimulation amplitude according to strength of input signal.

All reported aDBS systems in PD until now are based on automatic amplitude modulation (AM). AM can be applied in different designs. *ON/OFF AM* is an aDBS paradigm that varies between periods during which stimulation is given with a predefined amplitude and a set frequency and pulse width, and periods during which stimulation is switched off (Fig. 4A). ON/OFF AM systems studied in akinetic‐rigid PD patients applied stimulation as long as the beta‐LFP recorded in the STN exceeded a certain threshold.^6^, ^28^ In contrast, ON/OFF AM systems studied in tremor‐dominant PD patients were designed to start a stimulation period several seconds before tremor reoccurs based on tremor prediction using machine‐learning algorithms.^46^, ^48^ Because tremor should not reoccur during stimulation, there is no feedback signal that identifies the end of the stimulation period. Recent research has shown that a stimulation duration of 30 seconds led to a ratio of stimulation time versus tremor‐free time off‐stimulation over 50% in one third of patients.^62^ Future research will need to clarify how ON/OFF AM can be implemented optimally for different PD phenotypes and different input signals.

When using ON/OFF AM, other details should be considered. First, a ramping onset, which increases the stimulation voltage from zero toward a predefined amplitude, can be used to overcome paresthesia.^6^, ^32^ Furthermore, ON/OFF AM can be applied in a phase‐dependent manner, in which a stimulus is applied with a fixed latency to an input signal.^25^ Phase‐dependent aDBS is hypothesized to have advantages over standard aDBS. Increased clinical benefit is suggested by targeting specific pathological neurophysiological phases in PD.^6^, ^32^, ^34^ Also, phase‐dependent aDBS might induce long‐lasting beneficial effects attributed to possible long‐term potentiation/depotentiation in the STN.^81^ Moreover, phase‐dependent aDBS reduced tremor severity and prevented breakthrough tremor while consuming less energy compared to cDBS.^82^ Studies assessing these suggested advantages of phase dependency in aDBS are required.

Other AM aDBS paradigms use a gradual or a continuous AM approach. *Gradual AM* increases or decreases the amplitude stepwise when the input signal is respectively higher or lower than certain thresholds (Fig. 4B).^5^, ^42^ Minimal and maximal stimulation amplitudes and the voltage change per step have yet to be defined. Recently, a gradual AM approach based on tremor power introduced two feedback loop computations. One slow loop gradually adjusted the amplitude baseline to prevent re‐emergence of diminished tremor, and one fast loop adjusted the actual amplitude rapidly to mitigate occurring tremor.^83^ This design will need to be reproduced, and the added benefit should be assessed. *Continuous AM* links every possible input signal to a corresponding preset output amplitude (Fig. 4C). Thus, the output amplitude inclines toward a parallel line of the input signal.^21^, ^22^

It is clear that stimulation parameter modulation can be applied in several ways in aDBS. At this moment, no research has been done to compare different approaches in general, or for specific phenotypes or input signals. In the next paragraph, we will elaborate on the clinical demands toward stimulation parameter modulation in aDBS per phenotype.

## Stimulation Parameter Modulation Demands per Phenotype

An optimally performing aDBS system should prevent overstimulation during periods with less symptoms, and it should increase voltage in a timely manner to minimize the duration and severity of symptomatic periods. Therefore, frequency of input signal evaluation can be an important difference in stimulation parameter modulation according to phenotype. This should be based on the frequency at which the monitored symptom is expected to fluctuate or reoccur after stopping or decreasing stimulation.

In tremor‐dominant PD, an aDBS system should ideally stimulate on “tremor control” level before the tremor actually occurs. Given that tremor fluctuates rapidly, the AM approach should rapidly respond to tremor reoccurrence in order to minimize tremor duration.

Compared to ON/OFF AM, a gradual or continuous AM approach needs more evaluation before stimulation reaches tremor‐control level. However, ON/OFF AM always stimulates with the preset voltage, which might cause overstimulation.

In developing an optimal AM approach, development of tremor prediction machine‐learning models is important.^48^ At the moment, most of these models are very accurate in scaling tremor severity rather than predicting reoccurrence.^84^ Future studies should analyze different evaluation frequencies, corresponding computational costs, and tremor reduction to compare the feasibility of different AM approaches.

In akinetic‐rigid PD, motor symptom fluctuations will be less frequent and less acute. Different input signal evaluation frequencies in aDBS for akinetic‐rigid patients have not yet been compared. Whether gradual or continuous AM is superior to ON/OFF AM in this group is dependent on improved symptom reduction and prevention of over‐stimulation when stimulating between zero and maximal amplitude.

Recent work on the modulatory effect of aDBS on beta‐LFP suggests ON/OFF AM to be better suited for akinetic‐rigid patients than gradual AM.^32^ They found a correlation between longer beta‐bursts (>0.6 seconds) and clinical impairment. Consequently, this implies that these longer beta bursts should trigger stimulation, and rapid anticipation and frequent evaluation of beta power is thus needed. Because of this required rapid anticipation, they prefer ON/OFF AM.

However, if the input signal follows the rhythm of akinesia and rigidity fluctuations, the input signal evaluation frequency could decrease, and a gradual AM approach might also be suitable and efficient. Whether this less‐frequent evaluation is feasible with STN‐LFP recordings has not been explored. At the moment, wearable sensors might have more potential to accomplish this compared to STN‐LFP recordings.

In aDBS for PD patients suffering moderate dyskinesia, these considerations were also addressed.^42^ The researchers observed aDBS transitions more frequently than expected based on clinical symptomatology. They suggested a slow ramping onset of stimulation voltage adjustments or alternative use of the triggering threshold, for example, a higher threshold or a two‐step threshold, in order to prevent too frequent stimulation parameter adjustments.

## Future Considerations

### Issues for Clinical Implementation

As discussed above, the research field on aDBS in PD is rapidly evolving (Fig. 1). In this section, we will highlight additional prospective issues that should be solved to realize a feasible aDBS system for chronic therapy.

Individual expectations and desires regarding an aDBS system can differ because of interindividual differences in the clinical course and personal coping strategies in PD patients. Individually tailored aDBS paradigms should respond to these factors, particularly aDBS systems that enable personal nuances in stimulation parameter modulation. However, aDBS in PD first needs a feasible standard system and stimulation parameter modulation, or one aDBS system per phenotype, before individualized fine‐tuning can take place.

It is plausible that each individual will start aDBS therapy with a calibration period, similar to cDBS therapy. Instead of a trial‐and‐error period trying different amplitudes, frequencies, or electrode contacts, the aDBS calibration period might try out different frequencies of stimulation parameter modulation, different threshold levels, or different voltage‐steps per modulation. Ideally, this process is automated by a self‐regulating algorithm.

### aDBS During Sleep

Although not discussed yet, a feasible aDBS system should consider the differences in patient preference and input signal during sleep. Akinetic‐rigid patients might consider stimulation at night as important, given that they suffer from rigidity at night and in the morning. Tremor‐dominant patients might need less stimulation at night because of a lower disease burden.

Regarding input signals, electrophysiological signals are influenced by vigilance state and therefore deserve different interpretation during sleep periods than during awake periods.^31^ Also, wearable sensors might be programmed with “sleep” or “rest” detection algorithms, which initiates a specific “sleep‐stimulation paradigm.”

### Monitoring of Nonmotor Symptoms and Side Effects

In general, current aDBS input signals are focused on motor symptom detection to evaluate the therapeutic effect. As discussed before, a first step toward personalized therapy can be to develop different aDBS approaches for the different main motor symptoms per phenotype. Future designs might expand the specificity per phenotype by considering nonmotor symptoms^85^ and potentially side effects caused by aDBS, like autonomic functions, dyskinesia, or speech deterioration. Including these features will make data analysis even more complicated. This future challenge requires complicated nuances that are out of reach for aDBS presently.

### aDBS Modulation Other Than Amplitude Modulation

Later aDBS systems might explore the use of different stimulation parameter modulations for specific clinical situations, for example, frequency modulation (FM). Possibly, stimulation parameters in bilateral aDBS could be evaluated and adjusted per side separately, tailoring aDBS per side.

Application of FM is hypothesized to contribute to tailored DBS paradigms.^86^ Three recent reviews on low‐frequency STN‐DBS described beneficial effects on freezing of gait, speech, and swallowing that did not respond to, or were caused by, high‐frequency DBS. However, beneficial effects could not always be reproduced, and low‐frequency stimulation sometimes led to worsening of cardinal PD symptoms.^87^, ^88^, ^89^ The effect of variable frequency stimulation, a paradigm interleaving high‐ and low‐frequency DBS,^90^ will be explored soon.^91^

Also, pulse‐width modulation might provide clinical benefit in certain situations. By exciting thin axon bundles belonging to the direct cortico‐subthalamic pathway more selectively,^92^ therapeutic windows may increase using shorter pulse widths, while using less energy.^93^, ^94^

### Battery Power Balance

aDBS may require less battery power for stimulation compared to cDBS. In contrast, more battery power may be needed for data processing and transferal, for example, by Bluetooth. There are several options to minimize the additional power needed by the pulse generator and to eventually make battery replacement less frequent. First, comparing the computational demands of various signal processing and machine‐learning approaches should minimize the required power.^16^ Second, the possibility to perform analyses on external devices or cloud‐platform solutions should be evaluated. Energy saved by outsourcing these computations should be compared with the energy required of wireless data transfer. Third, rechargeable pulse generators should be further developed regarding clinical applicability.^95^

### Socioeconomical Relevance

We suggest that implementation of aDBS systems in PD care will result in beneficial socioeconomic effects. Most important, if aDBS results in an improved ratio between beneficial and side effects, quality of life will improve and patients will function better in society. Also, economic burden will decrease, given that PD patients can be part of the working population for a longer period and need less care.

## Conclusion

Although impressive progress in aDBS for PD has been made over the last decade, major challenges to chronic application are still pending. We believe that research into clinical associations of input signals should concentrate on different PD phenotypes. Because the correlation of different input signals with PD symptomatology varies (Fig. 3), we believe that no single currently available input signal will cover the heterogeneity of all phenotypes in PD patients. To achieve this ambition, thoughtful combining and selection of input signals is inevitable. The increasing trend of combining knowledge between neurologists, neurosurgeons, engineers, and computer scientists is crucial in this field and opens the gate to translational medicine 2.0: “from byte to bedside.”

## Author Roles

(1) Research Project: A. Conception, B. Organization, C. Execution; (2) Statistical Analysis: A. Design, B. Execution, C. Review and Critique; (3) Manuscript Preparation: A. Writing of the First Draft, B. Review and Critique.

J.H.: 1A, 1C, 3A

M.H.: 1A, 1C, 3A

M.K.: 3B

M.J.: 3B

Y.T.: 3B

P.K.: 3B

## Financial Disclosures

Pieter Kubben received an unrestricted grant from Abbott. Yasin Temel received grants from stichting Weijerhorst and stichting Sint Annadal.
